# Scrutinizing the epigenetics revolution

**DOI:** 10.1057/biosoc.2014.22

**Published:** 2014-08-04

**Authors:** Maurizio Meloni, Giuseppe Testa

**Affiliations:** a School of Sociology and Social Policy, University of Nottingham, Law and Social Sciences Building, University Park, Nottingham NG7 2RD UK.; b Honorary, College of Social Sciences and International Studies, University of Exeter, EX4 4RJ, Exeter, UK.; c European Institute of Oncology, Via Adamello 16, Milan 20139, Italy.

**Keywords:** epigenetics, imaginaries, molecularization, plasticity, responsibility, social policy

## Abstract

Epigenetics is one of the most rapidly expanding fields in the life sciences. Its rise is frequently framed as a revolutionary turn that heralds a new epoch both for gene-based epistemology and for the wider discourse on life that pervades knowledge-intensive societies of the molecular age. The fundamentals of this revolution remain however to be scrutinized, and indeed the very contours of what counts as ‘epigenetic' are often blurred. This is reflected also in the mounting discourse on the societal implications of epigenetics, in which vast expectations coexist with significant uncertainty about what aspects of this science are most relevant for politics or policy alike. This is therefore a suitable time to reflect on the directions that social theory could most productively take in the scrutiny of this revolution. Here we take this opportunity in both its scholarly and normative dimension, that is, proposing a roadmap for social theorizing on epigenetics that does not shy away from, and indeed hopefully guides, the framing of its most socially relevant outputs. To this end, we start with an epistemological reappraisal of epigenetic discourse that valorizes the blurring of meanings as a critical asset for the field and privileged analytical entry point. We then propose three paths of investigation. The first looks at the structuring elements of controversies and visions around epigenetics. The second probes the mutual constitution between the epigenetic reordering of living phenomena and the normative settlements that orient individual and collective responsibilities. The third highlights the material import of epigenetics and the molecularization of culture that it mediates. We suggest that these complementary strands provide both an epistemically and socially self-reflective framework to advance the study of epigenetics as a molecular juncture between nature and nurture and thus as the new critical frontier in the social studies of the life sciences.

Who you are is written in both pen and pencil: things written in pen you can't change: that's DNA; but things written in pencil, you can: that's epigenetics. (*Reliv International*, promoting the soy peptide extract, LunaRich X™)

## Succeeding by Blurring: The Irresistible Rise of Molecular Epigenetics

Molecular epigenetics, the ‘next big thing' in the world of bioscience (Ebrahim, 2012), is a scientific success story that thrives in the ambiguity of its own definition. As to success, there can be little doubt about it: it is enough to look at the 10-fold increase, over the last decade, in the number of publications carrying ‘epigenetic' in their title (Haig, 2012). Only in 2011 the figure of publications in the field had reached the astonishing amount of several thousands, possibly up to 20 000 depending on the search criteria (Jirtle, 2012), and at any rate has continued to increase since then. Similar efforts aimed at computing the rise of epigenetics in terms of new networks, institutes, conferences, curricula and journals confirm the vertical growth of the field across the full range of academic indicators.

Within a few years ambitious large-scale projects, such as the International Human Epigenome Consortium (IHEC) or the NIH Roadmap Epigenomics Mapping Consortium, aiming at mapping human epigenomes for a variety of cell types and/or disease states, have been launched worldwide. New journals (*Epigenetics, Epigenetics and Chromatin, Clinical Epigenetics*), professional bodies (the Epigenetic Society, the Clinical Epigenetics Society) and research centers have also appeared in just a decade. In sum, epigenetics has provided “a banner under which a new scientific movement has advanced” (Haig, 2012, p. 15). Even beyond the boundaries of biomedicine, various other disciplines have started to signal the impact of epigenetics on some of their fundamental tenets: from bioethics (Dupras *et al*, 2012) to human geography (Guthman and Mansfield, 2013), from political (Hedlund, 2012) to legal theory (Rothstein *et al*, 2009), from epidemiology (Relton and Davey Smith, 2012) to the philosophy of identity (Boniolo and Testa, 2011).

Unsurprisingly, even a cursory glimpse into popular media reveals the increasing stronghold of epigenetics also on public imaginary. Epigenetics has gone pop (Davey Smith, 2012) occupying the cover of global magazines under sensationalist claims such as “victory over the gene” (*Der Spiegel*, 2010) or “your DNA isn't your destiny” (*Time Magazine*; see Cloud, 2010). Holistic medicine and various spiritual advices are being reframed in epigenetic terms (Church, 2007). Perhaps unsurprisingly, a new market niche has also started to emerge with companies spinning the business potential of the epigenetic idiom, as exemplified in the case of Reliv International, a producer of nutritional supplements, that launched its latest soy extract under the banner “You to SuperYou: Direct Your DNA Naturally Through Nutritional Epigenetics” (reliv.com/lunasin-and-epigenetics).

## An Epistemology of the Imprecise

Precisely as a field, however, epigenetics seems to flourish in the remarkable ambiguity of its defining term, with its apparent ability to accommodate – and productively align – a rather diverse range of biological questions and epistemic stances. Echoing Rheinberger's (2003) endorsement for an ‘epistemology of the imprecise', we argue that the ability to entertain multiple understandings of what constitute epigenetic phenomena, and hence multiple ways to secure epigenetic evidence, is foundational to epigenetics' rise, both as a scientific discipline and as a popular phenomenon. Expanding on the notion of ‘boundary object' (Star and Griesemer, 1989), Rheinberger (2003) framed the gene as a boundary object that molecular biology has been gradually encasing within an eminently flexible boundary concept, thus supporting the claim that ‘boundary objects require boundary concepts' because, “as long as the objects of research are in flux, the corresponding concepts must remain in flux, too”. The same we believe applies today to epigenetics, with its elusiveness (Dupré, 2012), polysemantic nature (Morange, 2002, p. 56) and coexistence of multiple accepted meanings for some of its basic features (Haig, 2004; see also Bird, 2007 and Ptashne, 2007).

In what follows, we thus start out not with the aim to provide a full disambiguation of epigenetics (including its more recent – omic descendant *epigenomics*, in which epigenetic regulation is studied at the level of the entire genome), as this would be at this stage largely futile and indeed counterproductive. Rather, we find it useful to trace the contours of this eminently flexible concept (epigenetic) and of the versatile fields that its flexibility propels. Specifically, our first goal is to highlight some key junctures at which the diverse streams of epigenetic research collide as well as the main knots through which they become entangled or conflated. The reason is that these instances of epistemic blurring open for social theory unique entry points to engage with the potentially transforming aspects of this burgeoning field.

## Sources and Boundaries of Epigenetics

Epigenetics has a long history in biology, and its current molecular reconfiguration is the result of a series of conceptual and experimental shifts. The notion of epigenetics was first coined by embryologist and developmental biologist C. H. Waddington (1905–1975) in the 1940s to define in a broader *non-molecular sense* the “whole complex of developmental processes” that connects genotype and phenotype. “It is convenient to have a name for this complex” Waddington writes, and “ ‘epigenotype' seems suitable” (reprinted in Waddington, 2012). Note as an aside that the neologism *epigenetics* was coined by Waddington as a derivative of epigenesis (Van Speybroeck, 2002), that is in a developmental sense, and was not meant in the current popular sense of what goes beyond/upon (*epi –* in Greek) the gene.

A second parallel origin of the concept seems to have had a stronger influence on the present understanding. This second tradition originates with Nanney's (1958) paper, *Epigenetic Control Systems*, and refers more specifically to the expression of genetic sequences (Haig, 2012; Griffiths and Stotz, 2013). As Haig explains, in Nanney epigenetic control refers to “which volume in the library of genetic specificities was to be expressed in a particular cell”. It is this second, more squarely molecular meaning that resonates to a greater extent with contemporary practices and that we refer to as ‘molecular epigenetics' to differentiate it from the original, developmentally centered and broader Waddingtonian sense (see for a distinction, Griffiths and Stotz, 2013). In turn this ‘library-scanning' view is itself broad enough to accommodate two only partially overlapping meanings of molecular epigenetics.

On the one hand, in fact, the library analogy forms the backbone for today's broader – and in some respects more shallow – understanding of molecular epigenetic, where the ‘epi' has come to refer to virtually all levels of cellular function that overlay genes while representing the result – or indeed the cause – of their differential expression in different cells and/or in different conditions. This operational definition includes the full complement of chromatin (that is, the three-dimensional mesh of structural and regulatory proteins within which most DNA metabolism takes place) but also the transcriptome, the proteome and the various omic – slices into which life's complexity has come to be parsed along the biochemical classification of its constituent molecules. In this sense, Nanney's definition, at its broadest, translates epigenetics into a problem, or rather into *the* problem of gene expression, and depending on the level at which one chooses to analyze the latter, the former becomes more or less distant from its original physical link to the genome.

The second, more precise and demanding meaning in molecular epigenetics involves operational definitions that are mostly negative, as in the study of “any long-term change in gene function that persists even when the initial trigger is long gone that *does not involve* a change in gene sequence or structure” (McGowan and Szyf, 2010, p. 67 our italics), or of a “phenotypic variation that is not attributable to genetic variation” (Champagne, 2010, p. 300), or of that portion of phenotypes that is transmitted though cell division or organismal reproduction but that is not encoded in DNA. In all evidence, we are still fully within the library analogy, except that now the only volumes that count are those that remain open long after the first reader is done with them.

It is apparent that both meanings of epigenetic deflate the role of genes as causally privileged determinants of phenotypes, the former by emphasizing the regulatory context that extracts diverse functional outputs from the same genome, the latter by highlighting those instances in which non-genetic changes persist, either in time or in space or in both. Viewed from this angle, both strands of epigenetic thinking and experimenting are contributing to a style of thought that, following in particular Griffiths and Stotz (2013), we can define as postgenomic. In the

postgenomic era, when complete genome sequences are available for an increasing range of organisms, the range of molecular actors has expanded greatly. The genome is not merely a collection of genes, but houses diverse other functional elements. Genes no longer have a single function closely related to their structure, but respond in a flexible manner to signals from a massive regulatory architecture that is, increasingly, the real focus of research in ‘genetics'. (Griffiths and Stotz, 2013, p. 2)

Importantly, here postgenomic and postgenomics are meant not only *chronologically* (that is, what has happened after/*post* the deciphering of the Human Genome in 2003) but also *epistemologically*, as the recognition of those gaps in knowledge and unforeseen complexities surrounding the gene (Maher, 2008) that have made our understanding of its function cautiously provisional and perennially contingent.

Increasingly, it is under the overarching umbrella of epigenetics (in the first, more shallow meaning that we have sketched above) that the disentanglement of these new complexities is expected to take place, promoting a conflation of the *epigenetic* with the *postgenomic* around the context-dependent view of the gene (Keller, 2000; Oyama *et al*, 2001; Moss, 2003; Robert, 2004; Mameli, 2005; Morange, 2006; Stotz, 2006, 2008; Stotz *et al*, 2006; Griffiths and Stotz, 2007, 2013; Nowotny and Testa, 2011). In this contextual view, genes are addressed as “catalysts” more than “codes” (Elman *et al*, 1996), “followers” rather than “leaders” (West-Eberhard, 2003), “embedded inside cells and their complex chemical environments” that are, in turn, embedded in organs, systems and societies (Lewkowicz, 2011). As Meaney emblematically writes:

the function of the gene can only be fully understood in terms of the cellular environment in which it operates. And the cellular environment, of course, is dynamic, changing constantly as a result of signals from other cells, including those that derive from events occurring in the external environment. Ultimately, function can only be understood in terms of the interaction between environmental signals and the genome. (2010, p. 48)

Expectedly, this way of thinking about biological processes has major consequences for established dichotomies of twentieth century biosciences, and in particular for the genotype/phenotype distinction (coined by Johannsen in the 1910s). In the context of the gene-centrism of the modern evolutionary synthesis, the relationship between genotype and phenotype was typically thought of as a relationship between a cause and its visible and mechanistically deduced effects, “between a plan and a product” (Jablonka and Lamb, 2005, p. 33). In that theoretical framework the chain of causal links moved unidirectionally from the active genotype to the ‘dead-end' phenotype. In the postgenomic era, instead, the relationship between genotype and phenotype is more often represented, rather than as a linear causal chain, in terms of a “rope” (Griesemer, 2002), a term that wishes to capture the profound intertwinement of the actual genetic material with the various layers of its phenotypic “appearance” (Oyama *et al*, 2001). Surfing over this rope, epigenetics resumes its original Waddingtonian emphasis, becoming a convenient heading for the multiple strands and complex apparatus of “developmental transformations intervening *between* genotype and phenotype” (Pigliucci and Muller, 2010, p. 308, our italics; see also Schlichting, and Pigliucci, 1998; Robert, 2004; Hallgrímsson and Hall, 2011).

Pulling together the threads of these imbricated, blurred or at times frankly competing understandings of epigenetics, we can thus posit that its current and unifying thrust is, in a nutshell, the *promise to capture the analogical vastness of the* ‘*environmental signals*' recounted above through the digital representation of their molecular responses. If what seemed irreducibly analogic (the social, the environmental, the biographical, the idiosyncratically human) needs to be overlaid onto the digital genome of the informationally ripe age in a dyadic flow of reciprocal reactivity, then it seems that this overlay can succeed only once the analogic is interrogated, parsed and cast into *genome-friendly, code-compatible digital representations* (RNA, DNA found associated to specific chromatin modifications as in chromatin immunoprecipitation or ChIP, methylated DNAs etc.). In this respect, epigenomic profiles (transcriptomes, chromatin maps and the further bits of living matter that technology is progressively digitizing, from proteomes to metabolomes etc.) are increasingly fulfilling, in today's biology, the role that cellular lineages took on in what Morange refers to as the ‘crisis of molecular biology' in the 1970s and 1980s. Following the spectacular dissection of the genetic code, the challenge to explain development in equally molecular and code-compatible terms proved rapidly a major one. As Morange notes,

The roots of the crisis should be sought at the epistemological level: what molecular biologists cruelly lacked, what led them to a feeling of decadence, was the total absence of a definition of … what would be an explanation of development, [ … for this … ] required that another level of description of the biological facts not be discovered, but valorized. This level was the cellular level, and this explains the dramatic development of cell biology during these years. Cell biology provided what Harold Kincaid called the ‘place holders', the terms which are introduced to designate an entity, a process for which we have good evidence, but whose precise nature is unknown. (Morange 1997, p. 390)

Similarly, we argue that epigenomic profiles, in their expanding variety, provide the new place holders to anchor the environment to the genome and enable the attending analogic–digital translations, conceptually as much as experimentally.

Thus, having briefly mapped *the blurred and thereby productive* boundaries of today's epigenetics, we move now to explore three research pathways for an emerging field of ‘epigenetics and society': (i) epigenetic vistas across controversies, hypes and sociotechnical imaginaries; (ii) epigenetics between facts and concerns; (iii) the emergence of a new molecular materialism mediated by the instruments and classifications of epigenetic research. Recent studies have begun to chart the contours of the new social studies of epigenetics, from an inquiry into the attitudes of epigenetics researchers (Tolwinski, 2013) to an articulate endorsement of the possible types of engagement between epigenetics and the social sciences, ranging from the more ‘interventionist' to the more ‘self-reflexive' streams of Science and Technology Studies (STS) (Pickersgill *et al*, 2013).

Here we advance this agenda further by providing three critical elements: (i) a methodological anchor to the epistemology of the imprecise, which positions epigenetics *vis-a-vis* both its scientific antecedents (chiefly molecular genetics) and its prospective partner disciplines within the social sciences; (ii) a focus on the digital feature of current epigenetics as a key resource to trace its explanatory success, again *vis-a-vis* its antecedents and prospective partners; and (iii) a tripartite research program that should hopefully foster the exercise of a rigorous ‘political-epistemology', for which the focus on epigenetics provides a paradigm of the inherently socio-political nature of biological discourse.

### Pathway 1: Epigenetic vistas across controversies, hypes and sociotechnical imaginaries

The very notions of a “decade of the epigenome” (Martens *et al*, 2011) or even of an “era of epigenetics” (Hurd, 2010) reveal how rapidly epigenetics has been rising to that level of salience, in both scientific and societal imaginary, that warrants the dedication of defined timescales in public attention and investment. And despite the fact that this ‘decade' has just begun, it is not too early to reflect on the societal impact of epigenetics. As we have already seen in the past for genetics, neuroscience or stem cells, often pioneering but preliminary findings are construed as providing evidence upon which to draw consequences for human health and well-being, especially by policymakers, media commentators, life-style advisers and sometimes natural and social scientists themselves.

Indeed, there is the feeling that is already happening for epigenetics: in popular books epigenetics has already been employed to make claims about human talent (Shenk, 2010), and in scientific articles epigenetic markers have been used to make claims about social inequalities and race differences in health (Kuzawa and Sweet, 2009; Wells, 2010; McGuinness *et al*, 2012). In triangulation with findings from the Developmental Origin of Health and Disease (DOHaD) literature, epigenetic claims have been used to target mothers as a new center of responsibility (Paul, 2010), recasting the maternal body into a sort of “epigenetic vector” (Richardson, forthcoming). Other expectations, as we will review later, exist with regard to epigenetics providing a possible new ground for legal claims and extended notions of responsibility.

A social study of epigenetics therefore should start from a reflexive analysis of the way in which epigenetic knowledge is becoming “a social phenomenon in itself” (Landecker and Panofsky, 2013), including the imaginaries and visions that are catalyzing this transition, and that we will refer to here as *epigenetic imagination*. Its analysis will provide social scientists with a vast repertoire of empirical sources where to observe the full thickness of science and society interactions through three of the most flourishing streams of research in Sociology of Scientific Knowledge (SSK) and STS: (1a) the sociology of scientific controversies; (1b) the sociology of hypes and expectations; (1c) the emergence of sociotechnical imaginaries.

#### Controversial knowledge

The study of scientific controversies has been a key heuristic methodology in SSK and STS for more than three decades now, prompting analysts to focus on the way in which scientific disagreement is handled, on the resources and practices that allow disputes to arise and persist, and finally on the decisional mechanisms by which consensus is reached (Nelkin, 1979, 1992; Engelhardt *et al*, 1987; Brante *et al*, 1993; Martin and Richards, 1995; Roosth and Silbey, 2009; Martin, 2008). Precisely through the blurred and at times frankly competing epistemologies that underpin the classification of its phenomena, epigenetics offers no shortage of controversies, especially around the following themes: (i) the relevance of intergenerational inheritance of epigenetic traits especially in higher organisms; (ii) the reappraisal of the concept of gene, and of the assessment of its functional significance, in the light of the unforeseen extent of several *epi-*layers of regulation (as most vividly captured in the heated controversies over the universe of non-coding RNAs unearthed by the ENCODE Project (Doolittle, 2013; Graur *et al*, 2013)); (iii) the tension between the Modern Evolutionary Synthesis as a settled canon and the renewed interest, much more vocal than in the past, in epigenetic, neo-Lamarckian mechanisms of inheritance (Jablonka and Lamb, 2005); and (iv) the epigenetic underpinnings of human behaviors.

Here we briefly focus on two such controversies whose implications appear particularly far-reaching for the strands of sociological inquiry that we pursue in this work.

The first is a display of semantic tension more than an actual controversy, but illustrates nicely the contentious potential of the blurring that we have previously hailed as a key factor in epigenetics' success and the ambiguity of the epistemic space in which epigenetics prospers today. In a recent popular publication Eric Nestler, Director of the Friedman Brain Institute at the Mount Sinai Medical Center in New York and co-author of a very much quoted study on the epigenetics of psychiatric disorders (Tsankova *et al*, 2007), expresses caution on the potential of epigenetics by claiming that “much more work is therefore needed before we will know the extent to which epigenetic mechanisms represent *a third factor – beyond nature and nurture –* in controlling an individual's traits in health and disease” (Nestler, 2013).

For Sweatt (2013) instead, one of the leaders of the emerging field of neuroepigenetics, “it is now clear that there is a dynamic interplay between genes and experience, a clearly delineated and biochemically driven mechanistic interface *between nature and nurture*. That mechanistic interface is epigenetics” (p. 624). The point here, however, is not so much about caution versus optimism. Rather, what counts for us is the radically distinct epistemic space in which epigenetics is recruited as an explanatory resource, by two authors who are both authorities in their field and have recently coedited an important publication on the epigenetics of regulation of the nervous system (Sweatt *et al*, 2013).

In what Keller (2010) has defined as the mirage of a space between nature and nurture, Nestler posits epigenetic mechanisms as a third factor that reaches beyond both, whereas Sweatt sees them as the interface that obliterates the space and dispels the mirage. ‘Beyond' versus ‘between': there lies the difference it would seem, and indeed it will be interesting for the analyst to see whether such semantic tensions end up propelling fundamental theoretical or experimental distinctions, or whether they will remain as just the rather innocuous legacy of that systematic blurring we have outlined above. In other words, it is conceivable that if epigenetic mechanisms are framed as distinct from both nature (a proxy for genes in such discourses) and nurture (a proxy for environmental triggers), rather than as the lens that illuminates the former through the latter (and vice versa) the very questions that end up being asked *may well differ significantly*, and with them also the host of attending experimental systems.

The second controversy concerns the difficulty in establishing the existence and relevance of transgenerational epigenetic inheritance in humans. Predisposition to colorectal cancer can be inherited through genetic mutations in several genes, including *MutL homolog 1, colon cancer, nonpolyposis type 2* (MLH1) and *MutS protein homolog 2* (MSH2). Some cases, however, were found to be inherited through epimutations in these two genes, that is to say in the abnormal methylation that ablated their function despite the integrity of their sequence, and they initially constituted the most striking example, documented in molecular detail, of transgenerational epigenetic inheritance in humans (Chan *et al*, 2006). Subsequent scrutiny, however, revealed that MSH2 methylation (the epimutation) was due to a genetic mutation in a neighboring gene (Ligtenberg *et al*, 2009). And also for MLH1, while the jury is still out, it is proving difficult to rule out upstream genetic causes and to unequivocally establish that the epimutation is itself inherited through the gametes rather than being simply triggered right after fertilization (Daxinger and Whitelaw, 2012).

Beyond the details of these fascinating cases, what emerges is the problem associated with the depth and breadth of the molecular gaze that informs current biology, with its skyrocketing ability to reveal more and more minute details but also with the attending challenge to define thresholds of epistemic significance for each of them (Nowotny and Testa, 2011). For in the age of so-called *next generation sequencing* (the term itself a testimony to the open-endedness of the whole pursuit), with genomes and epigenomes stretching like acres of naked nucleotides ready to be read and re-read with ever greater accuracy, proving a transgenerational epigenetic effect in the outbred human population, requires *de facto* that all possible genomic causes are excluded. And yet, the vaster the genomic space we wish to sample to that end and the more certain we wish to be of that exclusion, the digger we have to deep. Thus, these controversies are paradigmatic because they set the stage for probing, in the many similar cases that will undoubtedly follow, what comes to constitute epigenetic evidence in the first place, through which work of purification and through which ‘trial of strength' (Latour, 1999), be it material or statistical.

Orthogonally to this peer-to-peer debate among scientists, a second source of friction is already well-identifiable in the tension between the supposed consensus around epigenetic knowledge, as it is propagated in society, in the front page headlines and also in some social science literature, and its uncertain, speculative status within the scientific community itself. It is in this mismatch between what is established and what is at present a source of heated scientific dispute that speculative assumptions, inflated discourses and enthusiastic media promotion, in a word all that create hypes around the epigenetic imaginary, are likely to find fertile ground. This brings us to the second point, the visions and expectations generated by epigenetic knowledge as it circulates through society.

#### Cycles of hypes and expectations from the genome to the epigenome and back

As a test case for the sociology of expectations, epigenetic knowledge is also very well-positioned. A growing body of research over the last years has investigated the forward-looking dynamics of science and technologies and the ‘generative' role of expectations in “guiding activities, providing structure and legitimation, attracting interest and fostering investment” (Borup *et al*, 2006, p. 286; see also, Brown *et al*, 2000; Brown and Michael, 2003; Van Lente, 1993, 2012). Although expectations have always been important in the modern history of science and technology, this stream of research has emphasized how “hyperbolic expectations about the future have become more significant or intense in late and advanced industrial modernity” (Borup *et al*, 2006). This saturation with anticipations, visions and promises has already accompanied the rise of genomics (Hedgecoe and Martin 2003; Fortun 2005; Sunder Rajan, 2006; Martin *et al*, 2008; Tutton, 2011) and it is against this backdrop that we wish to situate the current climax of expectations surrounding epigenomics. Specifically, we find that the hypes accompanying epigenomics, mainly at the level of popular science but also in sections of the scientific community, rest on a bivalent understanding of its relationship with genomics: on the one hand *as a missing link that can succeed where genomics purportedly failed*, on the other *as a quantum leap enabled by the very success of genomics*. This is because epigenomics, as we briefly summarize below, is exploding at a specific and highly interesting phase in the cycle of expectations and promises of genomics itself (for the literature on hype cycles, Van Lente *et al*, 2013).

Following the relative disappointment for the slow pace of translation of genomic knowledge into clinical practice, genomics is in fact experiencing now a major come back driven largely by the unprecedented leap in our ability to sequence individual genomes. In a nutshell we can say that the newly found confidence in the genome as an explanatory resource for human traits (especially diseases) marks precisely the transition from the slightly abstract notion of the genome writ large coming out of the Human Genome Project (HGP) to the eminently concrete sequences of multitudes of individual genomes, individual not only in the sense that they come from individual beings but indeed, and increasingly so, from individual cells of the same being. From cancer (Burrell *et al*, 2013) to neurodevelopmental disorders (Poduri *et al*, 2013) to, indeed, healthy development (De, 2011), next-generation sequencing has brought the genetic heterogeneity of our cells back to the fore, thereby beginning to illuminate the truly unprecedented extent of our somatic mosaicism (that is, of the genetic differences found among cells of the same organism) and to propose for it an important role in a variety of conditions. Indeed, in an almost ironic twist, the very technology of epigenetic reprogramming (which allows to reset the epigenome of individual somatic cells and derive from them unlimited amounts of pluripotent stem cells which, among other things, greatly facilitates genome sequencing) is one of the most powerful approaches to probe the depth of our genomic diversity, both within and among individuals (Takahashi and Yamanaka, 2006; Abyzov *et al*, 2012). Against the backdrop of these developments, which together re-emphasize the importance of genomes as explanatory resource, we can then observe how it is an intersection of two discourses that upholds the bivalent relationship between genomics and epigenomics that we have recounted above.

On the one hand, to the extent that the admittedly naïve expectations over the immediate impact of the HGP have not been fully realised, epigenomics has progressed within a new promissory discourse where its findings are conceptualized as the “key ‘missing piece' of the etiological puzzle” and what will make justice of the promises of the now discredited “genocentric focus in our approach to human disease” (Szyf, 2011). Examples of such a discourse abound and inform much of the excitement over epigenetics in biomedicine (Feinberg, 2008; Choi and Friso, 2010; Petronis, 2010; Chadwick and O'Connor, 2013; Mill and Heijmans, 2013).

On the other hand, the ability to study both genomes *and* epigenomes *together* at unprecedented resolution has been inviting a different discourse where the former regains primacy in shaping the latter, from the emphasis on genetic mutations in epigenetic regulators that underlie an increasing number of diseases (Ronan *et al*, 2013) to the notion that somatic genetic mosaicism is not only widespread during development and aging but that it can itself affect “the epigenetic patterns and levels of gene expression, and ultimately the phenotypes of cells” (De, 2011). Clearly, depending on how far the pendulum swings toward the poles of these two discourses, one encounters a range of epistemic nuances, from the mutually exclusive attempts to replace the genome with the epigenome (or indeed vice versa) as explanatory resources, to the mutually reinforcing attempts to probe them in the increasingly visible circularity of their interconnections.

In this respect, and unsurprisingly, twin studies are proving to be an especially informative domain in which to flesh out the mutual reconfigurations of these two discourses. A source of permanent wonder throughout human history, twins have come to be a unique challenge and an equally unique opportunity once some of them ‘became' monozygotic, that is, once embryology and genetics led us to trace their identity to the sameness of cellular and genetic constituents, thus setting them apart from their ‘lesser' siblings that happened to share only a womb at a given time (that is, the same *context of epigenetic triggers*, in today's language, see Nowotny and Testa, 2011). The genetic identity of monozygotic twins, cast against the range of their phenotypic diversity, has thus become the most visible manifestation of the genome's insufficiency as sole or even main determinant/predictor for several human traits, offering for this very reason a unique entry point into the dissection of non-genetic contributions. In its proposed role of critical intermediate between genotype and phenotype or genotype and environment (along the many shifts we have encountered above), epigenetics has thus acquired increasing prominence in twin studies, as witnessed by what is arguably its most visionary and cogent pursuit, namely the Peri/Postnatal Epigenetics Twin Study with its systematic and prospective scrutiny of individual epigenetic variation in twin cohorts starting from birth (Loke, 2013), that in turn builds on the first systematic scrutiny of the epigenetic changes that accrue over the lifetime of monozygotic twins (Fraga, 2005).

Against this backdrop, the recent popular science book by Tim Spector, *Identically Different* (2012) becomes a powerful example precisely because Spector is both a leading scientist in twin studies (Professor of Genetic Epidemiology at Kings College and founder of the UK Twins Registry, one of the largest world collections on twins) and, in this case, a popularizer of epigenetic findings. The book opens with Spector's confession, “Until three years ago, I was one of the many scientists who took the gene-centric view of the universe for granted”, and proceeds to translate into popular culture the epistemic tensions of epigenetic research in its quest for the new paradigm that fills in *where classic gene-centrism has failed*. If twin studies ground in the genome only 35 per cent of the variance that accounts for a whole range of psychological and medical traits (Spector, 2012, p. 147), where should one look for the remaining unexplained variance, Spector's key argument goes, if not in epigenetics? The point, however, lies precisely in how that unexplained variance, the analogical vastness of environmental signals we have recounted above, is *being cast within the same digitally friendly language of maps, codes or blueprints* that enabled the gene-centric paradigm to rise in the first place. Just to quote an example from Spector (2012), the “religious susceptibility gene” remains steady, in this narrative, in the ambition to ground culturally sophisticated phenomena onto molecular codes, with the difference that these codes now take the form of flexible and hence reversible switches rather than fixed circuits (p. 107).

In sum, our conclusion is mixed. If one were to look in epigenetics for a radical disavowal of the digital primacy of the genetic language, she would be disappointed and might well conclude, following the famous dictum from the Italian twentieth century masterpiece *The Leopard* that “everything needs to change, so everything can stay the same” (Tomasi di Lampedusa [1958] 1960). If instead one looked in epigenetics for a defiance of genetic determinism that succeeds precisely by applying the same digital language but to include rather than to exclude context (environment, biography, lifestyle and so on), then she may more likely perceive the innovative thrust of the field.

#### Epigenetic imaginaries

A growing interest in the broader landscape where scientists operate as “cultural producers” or “sociocultural entrepreneurs” has characterized recent work in the social studies of science (Fujimura, 2003). The notion of imagination and imaginaries has been employed by several authors to emphasize the “historically inflected and socioculturally sedimented” context where scientific knowledge takes shape and “interpolates technical, biomaterial, political-economic, social, cultural, and ethical elements” (Fortun and Fortun, 2005). The way scientific discourses are embedded with other cultural discourses and contribute to trigger the imagination of scientists and society has been analyzed especially in genetics and genomics. In a slightly different meaning Jasanoff and Kim (2009) have introduced the notion of sociotechnical imaginaries to emphasize, in the context of a study on nuclear power, the “promotion and reception of science and technology by non-scientific actors and institutions” and national differences in “collectively imagined forms of social life and social order reflected in ( … ) scientific and/or technological projects” (p. 120). It is therefore in this context of renewed interest toward the imaginative/imaginary context of science that it is possible to suggest a third line of reflexive investigation on epigenetic knowledge, what we can name here the ‘epigenetic imaginaries'.

Epigenetics has shown in just few years to be a powerful imaginative tool. The profound impact of epigenetics on society and its symbolic landscape is exemplified by the rapid diffusion within the popular press, in pop science books (Francis, 2011; Carey, 2012) and documentaries (such as the BBC “Ghost in Your Gene” program or the more recent “The hidden life of our genes”) of a whole series of new foundational stories that seem to play the same function as Dora's case did for Freud, Little Albert for behaviorism and Phineas Gage has been doing recently for moral neuroscience. These truly “dramatic epigenetic pin ups” (Davey Smith, 2012) are constantly retold among the wider public to illustrate the social/historical relevance of epigenetics: from the ‘thrifty phenotype' of the DOHaD hypothesis to the impact of the Dutch Hunger Winter (1944–1945) on the lifespan, decades later, of people prenatally exposed to it (among which, we are told, Audrey Hepburn), from the consequences of the siege of Leningrad to the transgenerational effects of famine in the remote village of Overkalix, in North Sweden.

Also the more squarely experimental stories are shaping intensely contemporary imaginary, becoming true *topoi* in the genre: it is the case, for instance, of the switching on and off of the *agouti* gene in mice (through a methyl-rich maternal diet in gestation) that makes genetically identical offspring look phenotypically different, in coat color but, more importantly, in weight and susceptibility to disease (Waterland and Jirtle, 2003, 2004). The passage on to the second generation of such an effect also has become emblematic of the idea that not only a mother's but a grandmother's diet can have a profound impact on the health of the grandchildren, an idea popularized in a classic epigenetic slogan such as: “you are what your grandmother ate” (Pray, 2004). A similar iconic status, especially for its possible implications for social research, has been reached by Meaney's (2001b) study on how variations in maternal behavior of rats alter methylation patterns in the offspring and how these epigenetic alterations affect the next generation, but can be reversed by cross-fostering the pups to more “affective mothers”. Along with the study on glucocorticoid receptor and child abuse (McGowan *et al*, 2009), this study has been hailed as evidence of how social experience gets under the skin (Hyman, 2009), and this metaphor has traveled widely in the social science context and is today reinforced by a parallel notion of epigenetic effects going “into the mind” (Toyokawa *et al*, 2012).

Finally, the epigenetic imagination is also about novel metaphorical resources (Nerlich and Stelmach, 2013). These metaphors are sensibly different from the language that characterized the genetic landscape. The metaphors of epigenetics are meant to show reversibility where before there was stability (the ‘pencil's trait' that can be erased versus the pen, the epigenetic software versus the genomic hardware), a variation on the genetic script (epigenetics as the German umlaut that can change meaning to a word without changing its material succession of letters (Urnov and Wolffe, 2001), or epigenetics as a removable post-it, a mere annotation on the genetic script), the persistence of past experiences through generations (“a ghost in the genes”, a “cellular memory of past events”, a “nuclear time bomb in our genes”, a poison, a curse, a scar, a mark in the genes) or holistic view of biological processes (epigenetics as a “symphony” of elements, replacing the absolutist role of the gene as “the director of the play”, see Noble, 2006; Qiu, 2006; Francis, 2011), but also to reinforce a new language of programming (fetal programming, environmental and social programming and so on).

### Pathway 2: Epigenetics between fact and concern

A second crucial aspect in the emergence of an ‘epigenetics and society' research program concerns the possible political, legal and ethical implications of epigenetic research. Following in the footsteps of its HGP antecedents, also epigenetics has started to trigger its own share of studies on Ethical, Legal and Social Implications (ELSI).

There can be little doubt as to the relevance of the ELSI studies that were spurred by and within the HGP, in terms both of what they accomplished directly and of what they set in motion more broadly for a sociologically minded approach to developments in the life sciences. In this work, however, we set out a task for ourselves that is clearly distinct from a discussion of the ELSI of epigenetics and that we hope in fact will be helpful in steering it along innovative directions. The reason is that, even at its most sophisticated, in its very wording the ELSI idiom reveals deep-seated assumptions, often unintentional or at any rate unscrutinized, about the flow of innovation in knowledge-intensive societies. After all, when discussing the ELSI of something, the very emphasis on the *implications of* this or that betrays the underlying model in which technoscientific ingenuity precedes (in the softer version) or frankly drives (in the harder flavor) social innovation. The analytical task is thus parsed from the outset into a neat demarcation of objects: on the one hand science (whose epistemic nitty gritty is more often than not black boxed), on the other society (or its many proxies, from laws to publics, from regulations to markets and so on).

This, however, bears little resemblance to what by now four decades of empirical work in STS have been consistently showing, namely that in technologically complex, knowledge-intensive societies the actions of epistemic and normative ordering, and their results, are not only interconnected but indeed mutually constitutive of each other. The idiom of co-production (Jasanoff, 2004) has captured this symmetrical constitution with particular cogency, highlighting how, when such settlements are eventually reached, they end up establishing not only an epistemic but also a normative order. In Latourian terms (Latour, 2004), we thus propose that the second pathway for an emerging social study of epigenetics is the following: to define how matters of epigenetic fact have already become mobilized as matters of social concern and, vice versa, how matters of social concern are becoming matters of epigenetic fact, all the while keeping alert to how, by the same token, also matters of epigenetic concern can become matters of social fact.

Specifically, we anticipate two prominent directions of this mobilization: (i) the digitization of the environment, with its attending discourse of collective and individual responsibility, including the notion of transgenerational accountability; and (ii) the identification of epigenomically distinct subgroups/subpopulations aiming at objectifying in molecular terms disadvantageous conditions and/or unequal social structures.

#### Digitizing the environment: Plasticity, responsibility and purity

The digitization of the environment, and its impact on responsibility, cuts across the main line of tension in molecular epigenetics, that between stability and reversibility. On the one hand molecular epigenetics is what promises to unravel genome's openness to environmental influences, social factors and the biographical marks of personal experience, making visible in molecular detail its essence of ‘reactive genome', following Keller (2011) and more recently Griffiths and Stotz (2013). Almost by definition, this openness to the environment, in its broadest sense, invites the expectation of change, the notion that once the genome has been downgraded from the high citadel of causal primacy to the messy roundabouts of reactive developmental resources, biological fates become inherently reversible and porous to intervention. From the massive investment in epigenetic modifiers within drug discovery to the rising prominence of environmental epigenetics (in the flavor of either blessing or curse), much of current molecular epigenetics revolves around the promise of change. On the other hand, however, the more stringent epigenetic phenomena, and those that are triggering more widespread fascination, are those that typically resist change, those states that defy in their stability the inherent disruption of genome regulation associated to the cycles of reproduction in cells or organisms.

Here, the same factors (environmental or else experiential) that promise change are also those that can leave permanent imprints or even scars. This ambivalence (or more appropriately: dialectic) is evident in Meaney and colleagues' groundbreaking studies on the effects of maternal care on gene expression and neural development in rat pups (Meaney, 2001b; Weaver *et al*, 2004) that have acquired almost iconic status in the present exploration of the biosocial link, including a recent expansion of their work to the human brain (McGowan *et al*, 2009). These studies reflect this profound line of tension in epigenetic research, implicit in the very notion of plasticity (Malabou, 2008). The plastic brain and the plastic genome are those that can give form but also receive form from the outside: you can change your genes, but also your genes (that is, the way in which they operate) can be changed, insulted, permanently damaged (or improved) by environmental exposures. It is thus against the backdrop of this tension between passivity and activity that we can most productively situate epigenetics' intellectual program of molecularizing the environment in digital terms thus making its impact on living beings measurable, archivable and comparable.

Unsurprisingly, this digitizing epistemology, along with the technological frontiers that it discloses and stimulates, is entering as a powerful resource in a reconfiguration of individual and collective responsibilities. The increasingly visible plasticity of the epigenome supports the new postgenomic discourse in which the genome is understood as something malleable that can be trained and modified through an “extended practice” (Spector, 2012). ‘Practice' is key here, as it captures how the potential reversibility of epigenetic marks grounds the rationale for continuous intervention and/or maintenance that may safeguard their plastic and hence vulnerable states. Responsibility ensues thus in response to both implications of epigenomic plasticity: (i) on the one hand frailty and danger, with the call to protect one's own epigenome from external insults (be they related to lifestyle, occupational hazards, environmental pollutants and so on); (ii) on the other opportunity and resource, with the promise to change and improve upon one's endowment.

This dialectic spans both scholarly and popular literature, as well-illustrated in a recent *Time* article where epigenetics is presented to the broad public as bringing “both good news and bad”, the bad news being the vulnerability of the epigenome to wrong lifestyles (“eating too much can change the epigenetic marks atop your DNA in ways that cause the genes for obesity to express themselves too strongly and the genes for longevity to express themselves too weakly” (Cloud, 2010) and the good news being the newly recognized capacity “to manipulate epigenetic marks in the lab”, which means that scientists “are developing drugs that treat illness simply by silencing bad genes and jump-starting good ones”. In all evidence, what lies ahead, and is already starting to unfold, is a major expansion in the care of the self along Foucauldian lines (Foucault, 1988) and as concrete examples of this digitizing thrust begin to emerge, they will constitute a rich palimpsest of options for STS scrutiny. In particular, the co-productionist framework will allow to unpack how the processes of gathering, standardizing and certifying epigenetic evidence will align with political, legal and economic rationalities in bringing about new settlements (or possibly reinforcing existing ones) across some of the most persistent dichotomies that structure our reflection on the human experience: normal versus pathological (or enhanced), safe versus dangerous, natural versus artificial, individual versus collective.

But if the epigenetic digitization of the environment functions in the spatial reconfiguration of the body *vis a vis* various sources of environmental exposure (along with the power structures within which they materialize), no less momentous is the temporal dimension of its impact. Indeed, inherent to the very same intellectual project is the notion that the epigenetic body is at once inhabited by the traces of its past and seeded with traces of its future. And these traces can stretch not only over one's own lifetime or over one's own offspring's lifetime, but possibly over the lifetime of several following generations. Indeed, as we saw above, the transgenerational resilience of epigenetic states, especially when it comes to humans, remains at once a topic of intense research (including heated controversies) and the magnet of greatest public fascination through the emphasis on an epigenetically haunted body, as most iconically captured in the very title of the BBC documentary on epigenetics “Ghost in Your Genes” and its bold announcement that “The lives of your grandparents – the air they breathed, the food they ate, even the things they saw, can directly affect you, decades later, despite you never experiencing these things yourself”.

We see at play, in principle but increasingly also in practice, an *expansion of the concept of responsibility* that reaches well beyond the individual and her direct offspring, fostering *the materialization of new bonds among generations*. Indeed, precisely this aspect has already triggered the attention of bioethicists and legal scholars in reassessing the inter-generational impact of traumatic social events and forecasting how “Epigenetic effects caused by chemicals and other environmental agents may provide a new source of litigation and liability under the common law. Such litigation, especially when it involves second and third generation effects, would raise a number of novel challenges and issues” (Rothstein *et al*, 2009). What is interesting here is how the ideas of natural, normal and pure that have shaped the discourse on the genome as a collective resource in need of protection (as “heritage of humanity” characterized by a natural state, in UNESCO's wording) will map upon the epigenome when it comes to so-called *intergenerational equity*. We see already glimpses of such a one to one translation, as in the recommendation that “each generation should maintain the quality of the human genome and epigenome and pass it on in no worse condition than the present generation received it” (ibid.).

Yet, it is precisely the very notion of a ‘quality' of an epigenome that will likely become the terrain of both scientific and social controversies as we move from the already-challenging task of defining reference epigenomes as standards for the advancement of the field (that is, the core mandate of IHEC) to the even greater challenge of accommodating and indeed interpreting those standards in terms of collective political intervention (Dupras *et al*, 2012; Hedlund, 2012). “Each of us has far greater responsibility than we ever imagined!” claims a popular medical American website (www.drfranklipman.com/faqs-on-epigenetics/). Indeed, the most visible effect so far of this narrative of hyper-responsibilization is probably what emerges from the intense moralization of the maternal body and behaviors in the triangulation of epigenetic and DOHaD writings. Epidemiological studies linking the lifestyle (diet, smoking) of boys during puberty with the disease risk of their grandsons and in general the male line (Pembrey, 2002; Pembrey *et al*, 2005) may possibly relieve the pressure on mothers, it has been claimed (Shulevitz, 2012), but the maternal body and her lifestyle remain so far overwhelmingly central (Richardson, forthcoming) as a target of responsibility for harmful epigenetic consequences on the child's health.^1^

#### Epigenetics in social policy and public health discourses

The second axis of investigation regards the huge expectations placed on epigenetics in terms of social policy and public health. Biological arguments in social policy have a well-deserved history of being discredited as *ad hoc* justifications for natural inequalities, social hierarchies and the immutability of social structures. These arguments endemically reappear in the public arena as the recent polemics in the United Kingdom by a government policy advisor on “Genetics outweighs teaching” illustrates (Wintour, 2013). Biology keeps being seen as a form of destiny but clearly epigenetics may introduce a strong discontinuity with this stereotypical thinking. By pointing to a new relationship between biological and social events, in which the social assumes a causative role in shaping human biology to a degree unseen before (Landecker and Panofsky, 2013), molecular epigenetics may produce significant conceptual changes in the applications of biological findings to social policy strategies.

Indeed, epigenetics is already being used in the service of explaining the persistence, within specific groups, of long-lasting social/health issues, such as obesity, cardiovascular disease, mental health, but also poverty, inequalities, neglect and their dysfunctional perpetuation generation after generation. Kuzawa's and Sweet's (2009) study on race is a very interesting example of this reconfiguration in epigenetic terms of racial disparities in cardiovascular health in the United States. Here an epigenetic developmental model of black–white disparities is said to provide “a more parsimonious explanation than genetics for the persistence of cardiovascular disease disparities between members of socially imposed racial categories”. For the authors, epigenetics offers “an important set of mechanisms by which social influences can become embodied, having durable and even transgenerational influences on the most pressing US health disparities”(ibid.).

A second key example of a reconfiguration of social disparities in epigenetic terms comes from the empirical study of McGuinness *et al* (2012) on the correlation between socio-economic status and levels of DNA methylation in Glasgow. This study, based on blood samples of 239 people from Glasgow's poorest and most affluent areas, found that global DNA hypomethylation was associated with the most deprived group of participants. The association between social deprivation and lower levels of methylation (in turn associated with enhanced inflammatory status and associated disease risk) enabled to posit for aberrant methylation, and by implication for other epigenetic signatures as well, the potential as new biomarker of social adversities, neglect and poverty. Local newspapers greeted this study as “the beginning of an explanation as to why Scotland's biggest city has the unwanted title of ‘the sick man of Europe' ”. Furthermore, charities celebrated the research as “ ‘startling evidence' of the impact poverty can have on children before they have even left the womb, and warned that cutbacks to welfare provision would only worsen the damage” (Mclaughlin, 2012).

It may be too early to say but, in a near future, it is foreseeable that epigenetic findings will become increasingly relevant in social policy strategies, and are likely to be positioned at the crossroads of three axes: (i) first, the use of epigenetic findings to offer an ultimate bastion of *biological evidence* for social deprivations and inequalities (Miller, 2010) and influence specific political agendas (in this reproducing the impact and allure of fMRI studies in social policy in the last decade: Wastell and White, 2012). (ii) Second, to the extent that in epigenetic research social adversities, class inequalities and other societal factors operate *through the modification of biological endowments*, the deep-seated distinction between natural and social inequalities that has structured much political science as well as much policy work will become so visibly blurred so as to be open for a potentially thorough reframing (Loi *et al*, 2013). To catch this intellectual novelty a new hybrid terminology, beyond the nature/nurture divide (Singh, 2012; Nature Editorial, 2012), has already started to appear, from notions of “metabolic ghetto” and “maternal capital” (Wells, 2010) to “molecular biology of the social position” (Niewöhner, 2011). (iii) Third, epigenetics may drive the emergence of a discourse that identifies, at the local level, subgroups/subpopulations with different epigenetic marks (reflecting for instance their disadvantageous conditions). These potentially vulnerable/risky subpopulations and ‘permanently undermined' groups may thus become the target of a new epigenetic biopolitics. A possible and updated revival of soft or Lamarckian inheritance in social policy discourses, in which local contexts decisively affect the quality of the epigenome and traumas travel intergenerationally to become ingrained within a specific population, should not make us forget that in the past these Lamarckian views of inheritance have become a fertile terrain for intensely racist and eugenic discourses on, for instance, the *irremediable degeneration* of the germ plasm in unfavorable environments (as in the case of the anti-Irish writings of British Lamarckian eugenicist E.W. MacBride, see Bowler, 1984). Without implying that this is likely to happen today, social and political scientists need to be aware of the complex and often subtle nature of the implications that different notions of biological heredity may have when transferred to policy contexts.

### Pathway 3: Paradoxes of somatic materialism

In the last two decades the expansive success of the life sciences, from neuroscience to epigenetics, has extended its reach over much of what had been once reserved to the perimeter of ‘nurture', the vaguely defined but prestigious space where social and cultural influences were sovereign. Nurture has become today increasingly subject to the techniques of measurement, digitization and storage that are part of that molecularization of environmental and societal factors that is foundational to epigenetics' intellectual program. It is as digital representations of the environmental, social or biographical aspects of ‘nurture' that epigenomic profiles enable the molecular, and at times also experimentally tractable, understanding of living beings. Yet, while molecularization has already sparked an important debate in the social sciences over the last decade (Shostak, 2005; Beck and Niewöhner, 2006; Rose, 2007; Nowotny and Testa, 2011), epigenetics seems to herald a new stage that entails “a highly selective scanning of the socio-material environment in order to make snippets of it available for experimental work at the molecular level. The sociomaterial environment and increasingly everyday life itself is framed and ordered in terms of its effect on molecular processes in the body” (Landecker, 2011; see also Niewöhner, 2011).

The same emergence and contemporary diffusion of a term like “exposome”,^2^ coined by epidemiologist Wild (2012) to define “every exposure to which an individual is subjected from conception to death” although important in rebalancing the focus of medicine toward environmental factors, is symptomatic of a “certain ontological flattening” as Landecker and Panofsky (2013) claim, “by which different categories of things in the world are made equivalent by recasting them as different forms of exposure”. The suffix – ome in exposome reflects such a digitization of all forms of environmental exposure, from motherly love to toxins, from food to class inequalities, into a single unifying category and syntax. It is at this level, we agree with Lock (2012), that “epigenetic findings may well set off a new round of somatic reductionism because research is largely confined to the molecular level”. It would be inappropriate, however, to read this novel somatic reductionism as the next episode in a genealogically linear saga of the rise of modern scientific reductionism. Things are much more complex and in a way interesting in epigenetic research and the reductionism and materialism that we are witnessing today may be qualitatively very different from the one driven by genetics in the last decade of twentieth century.

Specifically, the paradox on which we want to call the attention here is that, differently from gene-centered twentieth century biology, it is precisely the current unprecedented deflation and openness to environmental factors of the postgenomic gene, with the subsequent collapse of the nature/nurture border, to produce this new stage of materialism and somatic reductionism with its singular profile. Here, there is a two-way movement that is worth exploring in detail.

On the one side, the more scientists explore the molecular meanderings of the genome, the more they meet “the many ties that link the individual body and its molecules to the spatio-temporal contexts within which it dwells”, as Niewöhner (2011) has aptly commented. Unsurprisingly, this notion that the line between the biological and the social has been erased to an unprecedented extent has been greeted in the social science and humanities. Representative of this attitude is an important recent article by Guthman and Mansfield that celebrates *environmental epigenetics* as fundamentally undermining “the boundaries [that are] often taken for granted between what is internal and what is external to the body, between nature and nurture, and between time and space”. “There is nothing about the body that forms a solid boundary – or threshold – between it and the external environment” they claim and “this interchange of environmental and bodily molecules suggests a transformation in what we mean by ‘nature' and ‘nurture' such that the lines between them are being erased” (Guthman and Mansfield, 2013, pp. 12–14). This extreme openness of the epigenetic body to the world's signals is certainly a major shift away from the mainstream lesson of twentieth century biology. The most common view of the body in twentieth century biology was derived from a Weismannian understanding of it as “a causal dead end” (Griesemer, 2002) that saw causation going unidirectionally from genotype to phenotype (otherwise referred to as the hard distinction between soma and germ line). The Weismannian body found an isomorphic reconstruction in Crick's (1958) central dogma of molecular biology, which “states that once ‘information' has passed into protein it cannot get out again”. This made the body (that is, the phenotypic level) a mere passive receiver of genetic information via the protein chain (or a ‘vehicle' of the genes, as in Dawkins' (1976) later speculations that followed the same tradition). Much more in the spirit of ecological traditions, or (if one is allowed) of the early twentieth century phenomenological notion of the body as embedded in its vital contexts, the epigenetic body brings the Weismannian body to an end.

On the other side, however, epigenetics' materialization of novel links between the genetic and the social, its making the body porous and permeable to the world *is exactly the channel* by which the capture of the body in molecular terms is made possible. The openness of the genome to the social is thus always on the verge of collapsing the social onto a mere source of differential genetic expression. This dialectic within postgenomic research is implicitly recognized by philosophers of biology Griffiths and Stotz (2013) (two unambiguous critics of reductionism) when they write how, in the current postgenomic and epigenetic landscape, the study of nurture is becoming “potentially as ‘reductionist' – that is to say, mechanistic – as research in any other areas of the molecular biosciences” (p. 5). What we want to emphasize here is the fact that, again with Griffiths and Stotz (2013), “a more epigenetic understanding of nature” goes together with “a more mechanistic understanding of nurture” and both these phenomena are a direct consequence of the fact that genes are today postgenomically defined “by their broader context” (p. 228).

This is the reason why we do not believe that in this context it would make sense simply to read epigenetics as a climax of the themes of twentieth century genetics. It is possible as Sarah Richardson claims that in epigenetic research “*genes remain very much at the center*” and very likely, as Richardson and Lock have argued, that a novel wave of reductionism is very much an effect of contemporary epigenetics, but the epistemic sources of this reductionism are *very different from* those of late twentieth century reductionism. Whereas in late twentieth century gene-centrism, from sociobiology onwards, we found an increasing attempt to expand the reach of nature into the field of nurture, here the somatic reductionism of epigenetics is the effect of an opposite epistemic claim: that neither nature nor nurture makes sense anymore, and everything is part of an integrated and blurred nature–nurture ontogenetic system (Meloni, 2013, 2014). The same notion of mechanism employed by Griffiths and Stotz has to be understood, following Bechtel (2008), in an integrative, *quasi*-holistic way as something that “recognizes the importance of the organization in which the parts are embedded and the context in which the whole mechanism is functioning” (p. 21).

Similar paradoxes appear when dealing with epigenetics as “the agent of resolution” (Keller, 2010) of the nature/nurture debate. On the one side molecular epigenetics is certainly a welcome challenge to the biologically untenable dualism of nature and nurture (Meaney, 2001a, 2010). In Galton's own terminology, the opposition of nature and nurture was supposed to distinguish “what one brings into the world at birth” versus “influences that act after birth” (Logan and Johnston, 2007). It is clear just from this simple definition how fallacious this dualism appears today, when we know for instance how several forms of prenatal exposures have a profound impact on phenotypes in adult life.

On the other side, however, if epigenetics certainly undermines the naïve separation of nature and nurture, at the same time, in breaking this fragile boundary around which much of the twentieth century episteme of the social and human sciences was constructed, it brings to light an entire new set of conceptual problems. To see this more clearly we need to contextualize epigenetic research in a broader transition in the life sciences that increasingly incorporates the space of culture into an evolutionary framework.

The collapse of the boundaries between the cultural and the biological was strictly avoided in a post-Weismannian division of labor between the ‘nature fortress' and the ‘nurture fortress', but is instead very much part of new intellectual trends in biology, from Developmental System Theory to Niche Construction that have extended biological inheritance so much to include extragenetic resources such as culture, or the symbolic system. In these trends, culture is not a biological adaptation in neo-Darwinian sense such as evolutionary psychology, or a meme to be studied on the fashion of a (narrowly defined) gene-centrism, but something that is taken much more seriously, as one of the four dimensions of evolution, itself structured as an inheritance system (Jablonka and Lamb, 2005). We sympathize with this theoretical approach but would just like to recall that precisely in response to this integrative neo-Lamarckian language the social sciences reacted in early twentieth century and constructed their autonomous episteme based on a hard separation of biology and culture (Stocking, 1968; Kroeber, 1917). The novel epigenetic language of extended extragenetic inheritance is likely therefore to be as provocative for neo-Darwinism as it will be for the social sciences and the humanities (Meloni, 2014).

## Conclusions

That epigenetics heralds a revolution, what we alluded to in the title echoing a recent popular book (Carey, 2012), has become such a tacitly accepted notion that it has escaped scrutiny almost entirely. Here we set out to scrutinize the key claims harnessed in support of this revolutionary narrative, in scientific and lay discourses alike, starting from a brief historical and epistemological reappraisal of the various strands of epigenetic thinking, often productively blurred in their distinctions or at times frankly competing with each other.

More than the hyped upheaval promised by popular literature, what emerges from our analysis is more akin to what Italian political theorist Antonio Gramsci (see Gramsci and Forgacs, 1988) famously referred to in the 1930s as “passive revolution”. According to his definition a revolution is passive when, far from being a radical break, it unfolds as a long-term process in which progressive and backward-looking forces coexist and overlap. It is passive (as in the case of the Italian Risorgimento) because it does not have the strength for (or may not even aim at) changing ‘the essential' and ends up thereby proceeding in a sort of limping way. And yet, despite an uncertain route in which vocal gestures end up often void or usher into bombastic but sterile statements, its impact can nonetheless prove revolutionary.

Without overdoing the analogy between political theory and science, we think that the Gramscian framework captures well the ways in which the ambition of molecular epigenetics innovates the current discourse on life while remaining loyal to the molecular gaze that has made it so productive and hence prominent in our society. In a nutshell, we have argued that that ambition is to tie the regulation of the genome to the digitization of the environment, bringing into relief the temporal dimension that this link invites (including its most far-reaching transgenerational instances). We have then proceeded to analyze how the pursuit of this ambition exposes the most salient lines of tensions of molecular epigenetics (from the epistemic to the normative), opening entry points for a sociologically minded scrutiny for which we have proposed three paths of inquiry that will hopefully help structure an early engagement of social scientists with this still-emergent field of the life sciences.

## Footnotes

1 As Richardson (forthcoming) acutely notes in three of the most classic experimental studies of epigenetic mechanisms (agouti gene in mice; season's influence in voles; licking/grooming in rats) the epigenetic modification is always introduced via the behavior or physiology of the mother.

2 As a National Institute of Environmental Health Science document (http://www.niehs.nih.gov/about/strategicplan/visionary-ideas/health-status/index.cfm) explains the notion of the exposome “replaces the chemical-by-chemical approach to finding causes of disease and includes endogenous and exogenous exposures”. The emphasis on this new concept is evident from the following lines: “Characterizing the human exposome represents a challenge similar to the HGP, which began when DNA sequencing was in its infancy”. See also, The Human Exposome Project at humanexposomeproject.com/. Two major grants on the exposome have been awarded by the EU in 2012.
